# The Phantom Platelet Problem: Unmasking Ethylenediaminetetraacetic Acid (EDTA)-Induced Pseudo-Thrombocytopenia

**DOI:** 10.7759/cureus.81211

**Published:** 2025-03-25

**Authors:** Enoch Chi Ngai Lim, Chi Eung Danforn Lim

**Affiliations:** 1 Research and Development, Specialist Medical Services Group, Earlwood, AUS; 2 NICM Health Research Institute, Western Sydney University, Westmead, AUS; 3 School of Life Sciences, University of Technology Sydney, Sydney, AUS

**Keywords:** edta-induced pseudo-thrombocytopenia, in vitro artefact, misdiagnosis, platelet clumping, thrombocytopenia

## Abstract

Ethylenediaminetetraacetic acid-induced pseudo-thrombocytopenia (EDTA-PTCP) is an artefact caused by EDTA-dependent platelet clumping in laboratory testing. Unrecognized cases may lead to unnecessary investigations, diagnostic confusion, and inappropriate medical management. We present a case of EDTA-PTCP, a condition that is still commonly overlooked in clinical practice. A clinical suspicion should always be raised when a drop in platelet count in the blood is identified among otherwise asymptomatic patients. To confirm the diagnosis, serial platelet counts on alternative anticoagulants, i.e., citrate or heparin, and blood smear studies to check for platelet clumping have to be performed. EDTA-PTCP awareness is vital to prevent patients from unnecessary medical procedures and investigations. Early recognition of this diagnostic artefact and its treatment using the appropriate diagnostic methods will help clinicians avoid misinterpreting laboratory findings. This leads to precise management of affected patients and reduces unnecessary patient anxiety, mismanagement, and healthcare costs.

## Introduction

Ethylenediaminetetraacetic acid-induced pseudo-thrombocytopenia (EDTA-PTCP) is a well-established complication in medical laboratory science. However, despite its clinical importance, it is still somewhat overlooked and does not receive the attention it deserves in clinical practice [1]. This may result in a misdiagnosis and, thus, inappropriate treatment, causing a healthcare problem [1,2]. This phenomenon is based on the fact that EDTA, widely used as an anticoagulant in blood sample testing, is responsible for the clumping of platelets, and therefore, the automated hematology analyzers show erroneously low platelet counts [3]. The reported prevalence rate of EDTA-PTCP varies and can be anywhere between 0.1% and 2% of hospitalized patients, and it may get as high as 17% for certain outpatient populations [4]. The percentage difference is likely related to the variability in the study populations (hospitalized vs outpatient), laboratory diagnostic methods, heterogeneity in study designs, and sample selection criteria. Since EDTA is the most commonly employed anticoagulant in full blood count tests, a situation of EDTA-PTCP being missed will inevitably cause unnecessary referrals to hematologists, inappropriate management decisions, and patient anxiety. Problems of EDTA-PTCP versus true thrombocytopenia can be solved only if we make a correct diagnosis by distinguishing the two and treating them accordingly.

## Case presentation

A European male in his early 50s scheduled a routine examination with his general practitioner (GP). He was asymptomatic, with no history of bleeding disorders, systemic or B-symptoms, easy bruising, or the presence of any bodily affliction. There was no relevant medical history, and he denied smoking, regular alcohol intake, or taking prescription and over-the-counter medicines or supplements.

**Figure 1:**
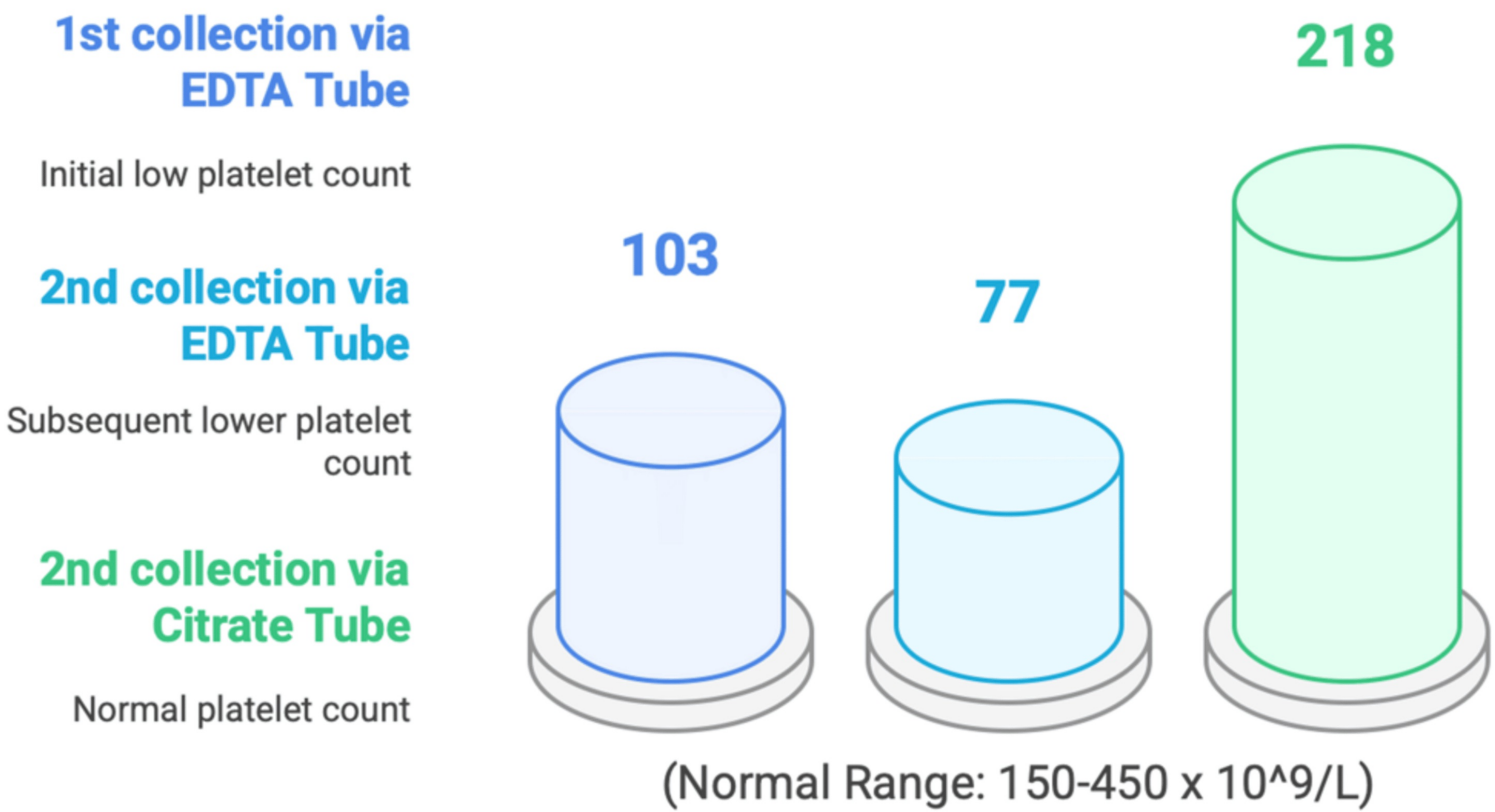
Platelet count measurements comparison

His physical examination was normal, and routine blood investigation was unremarkable except for the standalone finding of thrombocytopenia with platelet count noted at 103 × 10⁹/L (reference range: 150-450 × 10⁹/L). Realizing that the pre-analytical error might have affected the patient's results, the GP ordered a repeat Full Blood Count 20 days apart from the first test using an EDTA tube and a citrate tube to compare the findings. As shown in Figure 1, the follow-up free EDTA plasma samples revealed the platelet concentration has further decreased to 77 × 10⁹/L, while the citrate-anticoagulated sample showed a normal platelet count of 218 × 10⁹/L, which indicates an artefact of EDTA dependency. A peripheral blood film from the EDTA sample showed the clumping of the platelets, whereas it was absent in the citrate sample. The increased platelet aggregation was noted from the second EDTA sample compared to the first sample, leading to a further decrease in the platelet count. It is most likely due to the difference in collection time (the first sample was collected in the morning and transferred to the laboratory three hours after collection, while the second sample was collected in the early afternoon and transferred to the laboratory five hours after collection) and the delay in laboratory processing time.

## Discussion

Comparison with existing literature

This case corresponds to the literature that draws attention to the predominance and clinical significance of EDTA-PTCP as a frequently encountered but underappreciated laboratory artifact [1,2]. Like other cases documented previously, this patient was asymptomatic, with no underlying hematological disorder detected, reiterating the necessity of considering EDTA-PTCP as a cause of isolated thrombocytopenia without clinical signs of bleeding [5]. The finding of the difference between platelet counts of EDTA- and citrate-acidified samples and the presence of EDTA-dependent platelet clumping on a blood smear agrees with the literature [3]. This section will discuss the EDTA-PTCP prevalence, diagnostic confirmation, pathophysiology, and clinical implications. 

Prevalence and clinical significance of EDTA-PTCP

EDTA-PTCP is an in vitro artifact with a prevalence of 0.1% to 2% among hospitalized patients [6] and 0.1%-0.2% among the general population [7]. It is the result of the exposure of cryptic platelet surface antigens, including glycoprotein IIb/IIIa, which facilitates the binding of natural anti-platelet antibodies, causing the aggregation of platelets in specimens containing EDTA-anticoagulated blood samples [5]. Considering that the everyday clinical laboratory uses EDTA on an extensive range of hematological analyses, the undetected or ignored phenomenon may lead to unnecessary examinations, unjustified referrals, and inappropriate treatments.

Clinical suspicion and diagnostic confirmation

EDTA-PTCP should be suspected in asymptomatic individuals with isolated thrombocytopenia, especially if there is no overt clinical evidence of bleeding or other hematological disorders [2,3]. The case should be confirmed by repeating the platelet count using the alternative anticoagulant, which can be citrate or heparin [3]. These anticoagulants do not cause the platelet clumping, so an accurate, true platelet number is determined. In addition, a microscopic examination of a peripheral blood smear yields further diagnostic confirmation by featuring the platelet aggregates in the EDTA samples and such normal platelet distribution in the citrate samples [1].

Pathophysiology and role of blood smear examination

EDTA-PTCP's mechanism of action relies upon a conformational alteration of platelet surface glycoproteins resulting from exposure to EDTA. This alteration brings forward otherwise hidden epitopes and finally causes the bonding with anti-platelet antibodies [3]. Such antigen-antibody interference clumps platelets together, thus generating a misleading count of platelets in a cell counter employing automated technology.

It is important to know that a blood smear analysis is a critical way to tell the difference between EDTA-PTCP and secondary thrombocytopenia. Although true thrombocytopenia manifests as an overall drop in platelet counts, EDTA-PTCP is described by the compounds of the platelet aggregates [3]. A non-aggregating picture of a carotene sample with citrate as a coagulant can support the diagnosis.

Implications for clinical practice

Awareness of EDTA-PTCP is crucial to prevent inappropriate medical procedures, such as bone marrow biopsies, platelet transfusions, and hematology consultations [1,8]. Clinicians ought to detect this artifice by choosing relatively cost-effective and satisfactory confirmatory tests, such as repeat platelet counts in citrate or heparin tubes, to avoid the problem of misdiagnosis. By resolving this artifact, not only is diagnostic performance increased, but the patient's anxiety level and healthcare costs are decreased by reducing unnecessary investigations and treatments.

On the other hand, a failure in identifying EDTA-PTCP may prove fatal when it comes to the clinical ramifications, such as unnecessary workups, which are generally for immune thrombocytopenia or hematological malignancies, which may even cause anxiety among patients as the concerned health expenditure may increase [1]. Such cases of EDTA-PTCP misdiagnosis have been reported in the literature and have led to inappropriate treatment with corticosteroids, intravenous immunoglobulins, and even splenectomy [1].

In case of suspicion or confirmation of EDTA-PTCP, citrate is generally selected as the best alternative anticoagulant for the repeat platelet count determination [9]. Citrate can significantly reduce platelet clumping, provides better accuracy, and is easy to handle in the laboratory. Heparin is less likely to be used because it has the risk of analytical interferences and does not preserve the optimal cells. As a result, the precondition is that repeating platelet counts using citrate-anticoagulated samples after the first observation of EDTA-PTCP is currently regarded as the preferred clinical and laboratory practice [9].

## Conclusions

This case report aims to demonstrate the importance for clinicians to recognize EDTA-PTCP to prevent patients from undergoing unwarranted tests and receiving incorrect treatment. The variation in platelet counts between EDTA- and citrate-anticoagulated samples and the platelet clumping seen in peripheral blood film suggest that the resulting artifact can resemble real thrombocytopenia. Implementing a structural investigative and diagnostic approach will allow clinicians to rule out false-positive results and prevent patients from experiencing needless stress and invasive medical interventions. Through increasing the awareness of this condition, clinicians can achieve better diagnostic precision, safeguard patient safety, and enhance healthcare service delivery.
